# Using Wavelet Coherence to Characterize Surface Water Infiltration into a Low‐Lying Karst Aquifer

**DOI:** 10.1111/gwat.13012

**Published:** 2020-05-15

**Authors:** Philip Schuler, Èlia Cantoni, Léa Duran, Paul Johnston, Laurence Gill

**Affiliations:** ^1^ Department of Civil, Structural and Environmental Engineering Trinity College Dublin, Dublin 2 Ireland; ^2^ Irish Centre for Research in Applied Geosciences (ICRAG), Belfield, Dublin 4 Ireland

## Abstract

Karstified carbonate aquifers may receive significant recharge contributions from losing streams, hence, the knowledge about surface water‐groundwater (SW‐GW) interactions is crucial with regard to water management (e.g., source protection zone delineation). The dynamics of SW‐GW interactions may depend on factors such as the relative water levels between streams and aquifers, resulting in a temporal variation of exchange, which imposes complexity to the understanding of such dynamics. This study highlights the use of high‐resolution time series and multiresolution analysis to help to gain insights into such complex dynamics. Wavelet coherence is applied on hourly time series of rainfall, stream, and spring discharges of a low‐lying karstified spring catchment to yield a correlation in the time‐frequency domain. This analysis provides comprehensive information on the overall impact of the river on the spring, which is supported by the cross‐correlation function, as well as by more detailed information, including time‐variant influences such as a threshold level of influence. Field observations of turbidity sampling at the spring appear to support this interpretation. This innovative approach relies on basic hydrological parameters, water level, or discharge, and is therefore applicable to many other systems with such existing time series.

## Abstract 2

*Article impact statement*: Wavelet coherence yields high‐resolution information on surface water‐groundwater interaction.

## Introduction

The interaction between groundwater and surface water through a transition zone along streams (i.e., hyporheic zone) has been receiving increasing attention (Barthel and Banzhaf 2016; Conant et al. 2019), focusing predominantly on homogeneous and porous aquifers, as outlined by Bailly‐Comte et al. (2008). In the context of karst hydrogeology, surface water‐groundwater (SW‐GW) interactions can play a significant role in upland and lowland system dynamics, yet, the analysis of such a phenomenon is less common. Kalbus et al. (2006) differentiate four methodologies to quantify the site‐specific flux between surface water and the phreatic zone: (1) direct measurements of water flux (e.g., seepage meters), (2) heat tracer methods, (3) methods based on Darcy's law, and (4) mass balance approaches. However, the application of any of these methods may be misleading in the context of heterogeneous karstified rock with non‐Darcian groundwater flow (Giese et al. 2018) due to the inherent complex interactions that vary on a small scale and at a high temporal resolution. For example, the diurnal oscillations of surface water temperature can complicate the interpretation of heat tracer tests. Hence, the purpose of this research is to investigate additional tools that can be applicable for the assessment of SW‐GW interaction.

In the context of karstified limestone aquifers, river bed exfiltration may constitute significant aquifer recharge (Salvador et al. 2012; Smith et al. 2015; Koeniger et al. 2017) while the magnitude and direction of the interaction may be time variant depending on the hydrometeorological conditions (Bailly‐Comte et al. 2009; Charlier et al. 2015b; Chapuis et al. 2020). Linking (and delineating) surface water and groundwater “bodies” is a crucial aspect with regard to integrated catchment management and the protection of surface waters and groundwaters, which is a priority of the European Water Framework Directive (WFD) (European Commission 2000, 2006, 2012). Yet, the effective implementation of the directive has been slow (Voulvoulis et al. 2017), and with much of the surface waters still reported to be below the desired status “Good” (European Commission 2012; Deakin et al. 2016).

Methods applied to investigate SW‐GW interaction in karst, may be considered as rather qualitative or semiquantitative as most of the approaches are “indicator‐based” by relating chemical parameters observed in spring discharge, such as chlorophyll‐a fluorescence (Gaillard et al. 2020), nitrate and phosphate (Charlier et al. 2020), or stable isotopes (^2^H, ^18^O) (Binet et al. 2017) to surface water infiltration. Other approaches may use water level or discharge time series of streams and their related springs applying, for example, inverse modeling of lateral river inflows or correlation and spectral analysis (Bailly‐Comte et al. 2008). A limitation of the latter method is the time‐invariant information gained, which can be overcome by applying wavelet transforms (Labat et al. 2000b) to yield information in both, time and frequency. Wavelets show the spectrum of frequencies/scales contained within a signal and their change in time. The respective content of two time series can be compared more specifically using Wavelet coherence (WTC) which is a measure of the correlation between the two nonstationary signals as a function of frequency (Torrence and Compo 1998; Grinsted et al. 2004). Such information is particularly relevant with regard to highly dynamic and nonstationary fluxes related to SW‐GW interaction in karst. In hydrogeology, WTC has been used, for example, to identify relationships between climate patterns and groundwater levels (Holman et al. 2011; Tremblay et al. 2011). More specifically to karst hydrology, the use of WTC is innovative, and, to the knowledge of the authors, it is limited to characterizing seasonal or multiannual rainfall‐discharge responses or river runoff dynamics (Salerno and Tartari 2009; Chinarro et al. 2010; Charlier et al. 2015a; Huo et al. 2016).

The aim of this study is to introduce the use of WTC to study SW‐GW interactions between a losing stream and the receiving spring in time of a low‐lying karst system using discharge (or equivalently water level) as the standard monitored parameter. No a priori information about any relationship is needed, which makes this method suitable as a first investigation tool on a catchment or even at a regional scale (Barthel and Banzhaf 2016). The method was successfully applied in a low‐lying karst catchment in western Ireland along with event‐based hydrometeorological monitoring.

## Research Method

### Study Area

**Figure 1:**
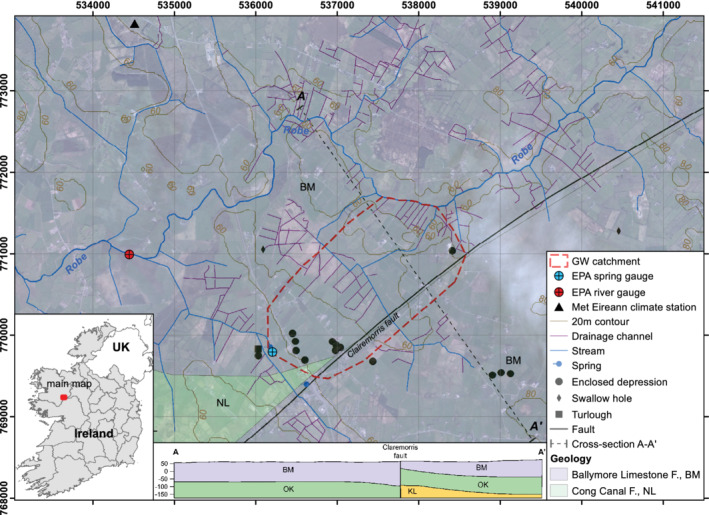
Geology of the Ballindine groundwater catchment and location of the MetEireann climate station at Claremorris.

Ballindine spring is located in County Mayo, western Ireland (Figure 1) which, prior to this research, had not been systematically studied from a hydrogeological perspective. Within the scope of the research, the catchment boundaries were delineated covering ∼3.3 km^2^ underlain by Lower Carboniferous limestones, of the Ballymore Limestone formation (BM), which consists of dark fine‐grained limestone and shale, and the younger Cong Canal formation (NL), which is a thick‐bedded pure limestone (GSI 2017). Older successions include the pale gray massive Oakport Limestone formation (OK) and the Tournaisian aged dark‐gray, fine‐grained argillaceous nodular Kilbryan Limestone formation (KL) that both include shale. The thickness of the limestones may reach several hundred meters. The location of the spring is presumably related to the regional Claremorris fault, which bounds the delineated catchment to the east and which seems to constitute a path for preferential dissolution impacting on the course of major streams, such as the River Robe (Figure 1). The general groundwater flow direction was unknown, as there are no known boreholes or observation wells within the study area.

The catchment elevations range between 48.8 m above sea level (masl) at the spring, <54.1 masl at the river bed, and up to a maximum of 65 masl within the catchment. Landuse across the study area is mostly agricultural grassland that is artificially drained by pervasive shallow channels intersecting the low‐lying topography and draining the top of the phreatic zone into the River Robe. The average annual rainfall for the hydrological years from 2010 to 2018 was 1265 mm (668‐mm effective rainfall) and the mean annual temperature was 9.3 °C.

### Data

Water level and discharge of the River Robe and Ballindine spring is monitored by the Irish Environmental Protection Agency (EPA) at 15‐min intervals (Figure 1). The data from both gauging stations was downloaded from the EPA HydroNet website (http://www.epa.ie/hydronet) and aggregated to an hourly time step using the software R (version 3.2.3) and the interface RStudio (version 1.1.453, www.rstudio.com). A data gap in the spring discharge time series between July 1, 2015 and November 20, 2015 was filled by using a simple rainfall‐discharge reservoir model (KarstMod, version 2.2.0.s) (Mazzilli et al. 2019) with a Kling‐Gupta efficiency of 0.89 during calibration (hydrological years 2012 to 2015) and 0.74 (hydrological years 2016 to 2018).

The monitored discharge of the Robe River comprises a contribution from the upstream Ballindine spring (although this only accounts for <1% of the total flow in the river). For all numerical analyses, the river time series were corrected from spring contribution by subtracting the latter from the former on the same time step.

**Figure 2:**
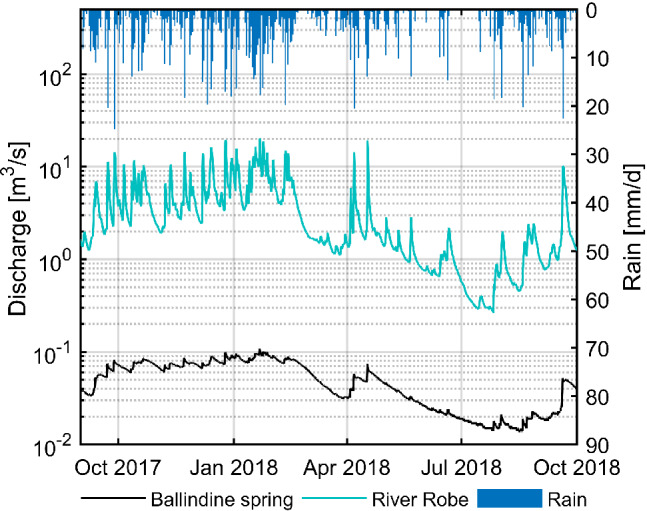
Hourly runoff and discharge of the River Robe and Ballindine spring and daily rainfall monitored at Claremorris during the hydrological year 2018.

Climate data used in this study comprises precipitation [mm/h] provided by the MetEireann station at Claremorris (Figure 1, 68 masl), 4.5 km north/northwest of Ballindine spring. The hydrological data covers the period of April 29, 2009 to October 1, 2018, exemplified for the hydrological year 2018 in Figure 2.

Event‐based sampling of turbidity was conducted for 10 d between January 30, 2019 and February 10, 2019 at Ballindine spring using a GGUN‐FL30 flow‐through field fluorometer (Albillia Co., Neuchâtel, Switzerland).

### Cross‐Correlation

In karst hydrology, cross‐correlation is frequently applied to characterize input–output relationships (Padilla and Pulido‐Bosch 1995; Angelini 1997; Larocque et al. 1998; Labat et al. 2000a; Mathevet et al. 2004; Massei et al. 2006).

For a discrete number of pairs of values (*x*_1_, *y*_1_), (*x*_2_, *y*_2_), …(*x*_*n*_, *y*_*n*_) it is shown that the cross‐covariance *c*_*xy*_(*k*) at lag *k* can be estimated by,

(1) $$c_{\mathrm{xy}}\left(k\right)=\left\{\begin{array}{c} \frac{1}{n}\sum_{t=1}^{n-k}\left(x_{t}-\bar{x}\right)\left(y_{t+k}-\bar{y}\right),k=0,1,2,\ldots \\ \frac{1}{n}\sum_{t=1}^{n+k}\left(y_{t}-\bar{y}\right)\left(x_{t-k}-\bar{x}\right),k=0,-1,-2,\ldots \end{array}\right.$$

And the cross‐correlation *r*_*xy*_ is given by,

(2) $$r_{\mathrm{xy}}\left(k\right)=\frac{c_{\mathrm{xy}}\left(k\right)}{s_{x}s_{y}},k=0,\pm 1,\pm 2,\pm \ldots$$

where $\bar{x},\bar{y}$ and *s*_*x*_*s*_*y*_ are the means and the standard deviations of series x and y, respectively.

The cross‐correlation function (CCF) measures the linear relationship between time series based on the assumption that the time series are regarded as bivariate stochastic processes that are stationary (Box and Jenkins 1976). The CCF between rainfall and spring discharge and between river and spring discharge was executed using the software R/RStudio (Venables and Ripley 2002).

### Wavelet Coherence

Given two signals *X* and *Y* with the wavelet transform $W_{n}^{X}\left(s\right)$ and $W_{n}^{Y}\left(s\right)$, a cross‐wavelet spectrum $W_{n}^{\mathrm{XY}}\left(s\right)$ can be calculated using $W_{n}^{\mathrm{XY}}\left(s\right)=W_{n}^{X}\left(s\right)W_{n}^{Y^{*}}\left(s\right)$ where $W_{n}^{Y^{*}}$ is the complex conjugate of $W_{n}^{Y}\left(s\right)$. The cross‐wavelet power is given as $\left|W_{n}^{\mathrm{XY}}\left(s\right)\right|$, and the coherence $R_{n}^{2}\left(s\right)$ is given by,

(3) $$R_{n}^{2}\left(s\right)=\frac{\left|\;S\;{\left({s^{-1}W}_{n}^{\mathrm{XY}}\left(s\right)\right)}^{2}\right|}{S\;\left(s^{-1}{\left|W_{n}^{X}\left(s\right)\right|}^{2}\right)\cdot S\;\left(s^{-1}{\left|W_{n}^{Y}\left(s\right)\right|}^{2}\right)}$$

ranging between 0 and 1 with the smoothing operator *S* and the circular standard deviation *s* (Grinsted et al. 2004). Confidence levels can be derived using the square root of the product of two chi‐square distributions (Torrence and Compo 1998).

**Figure 3:**
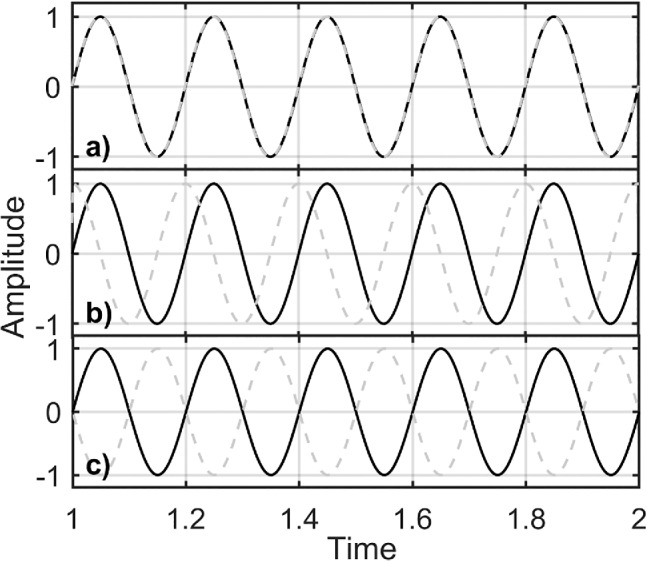
Examples of phases: (a) in phase (0°), (b) out of phase gray dashed line leading (30°), and (c) completely out of phase (180°).

The coherence is a localized correlation coefficient in time‐frequency space, hence, it is a measure of linearity of the relationship. Cause‐effect relationships can be assessed by evaluating the cross‐wavelet phase angle using the circular mean of a set of the angles and the confidence interval of the phase difference plotted in an angle between 0 and 360°. Two sine waves are in phase, if their phase difference is 0° (horizontal arrow pointing right), out of phase or completely out of phase if their phase difference is 180° (horizontal arrow pointing left) (Figure 3a–3c). Regions of high coherence, which further show a consistent phase relationship, that is, “phase locked,” or that change progressively and smoothly, are suggestive of a causality between the time series (Grinsted et al. 2004). A “cone of influence” can then be computed for the regions in which edge effects become important (Torrence and Compo 1998), and in which the interpretability of the results is reduced.

WTC (including mean phase angles) was applied between rainfall and spring discharge and between river and spring discharge using MATLAB R2016b (version 9.1.0., MathWorks Inc., Natick, Massachusetts) and the MATLAB toolbox developed by Grinsted et al. (2004).

## Results

### Cross‐Correlation

**Figure 4:**
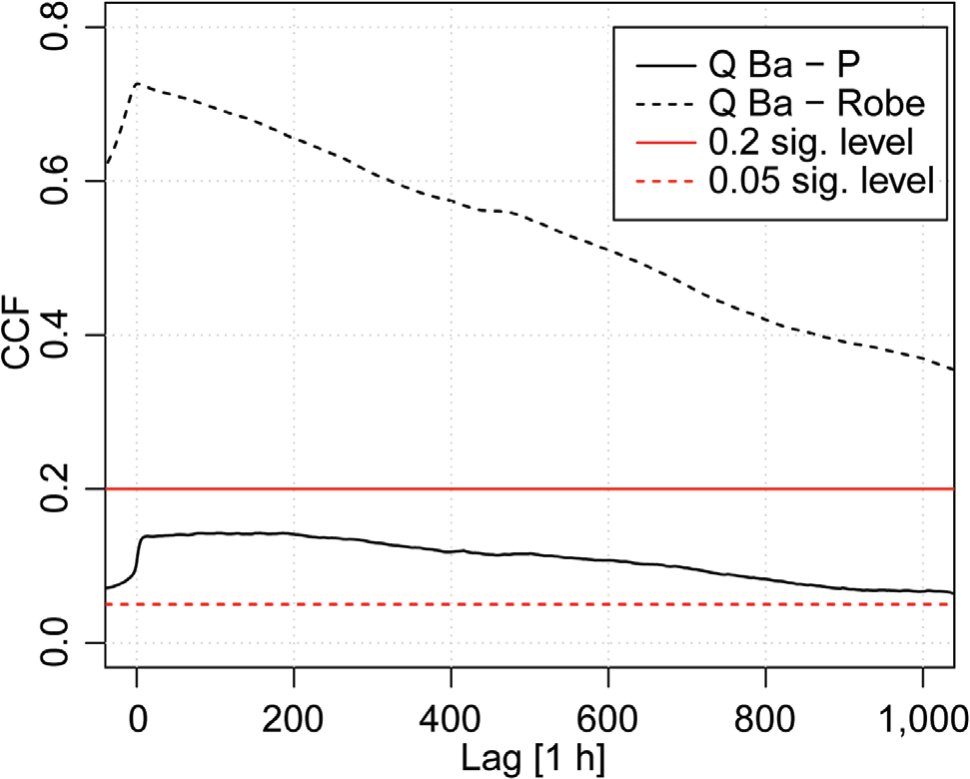
Cross‐correlation function between (1) the hourly discharge of Ballindine spring (Q Ba) and rainfall (P) (solid black line) and (2) the discharge of the River Robe (Q Robe) and hourly discharge of Ballindine spring (Q Ba) (dashed black line) between April 29, 2009 and September 30, 2018. The significance levels at 0.2 and 0.05 are also plotted for the QBa‐P and QBa‐Robe respectively.

The CCF was applied to evaluate the time‐invariant relationship between rainfall and spring discharge, and between the river and spring discharge (Figure 4). The results obtained show that the two relationships present contrasting (linear) behavior. The CCF between rainfall and Ballindine spring reaches the peak after a lag of 60 h at 0.13, indicating a relatively weak relationship. In turn, the CCF between the River Robe and Ballindine spring peaks with a lag of 1 h at 0.73. While rainfall is not autocorrelated, the runoff and discharge time series of the River Robe and Ballindine spring are both highly autocorrelated. Nevertheless, the relatively high linearity between the river and the spring (compared to the linearity between rainfall and the spring) may suggest a connection between river and spring as the time lag is short. Both lags are positive and hence, assuming a causality, the direction of influence is from the river/rainfall towards the spring.

### Wavelet Coherence

**Figure 5:**
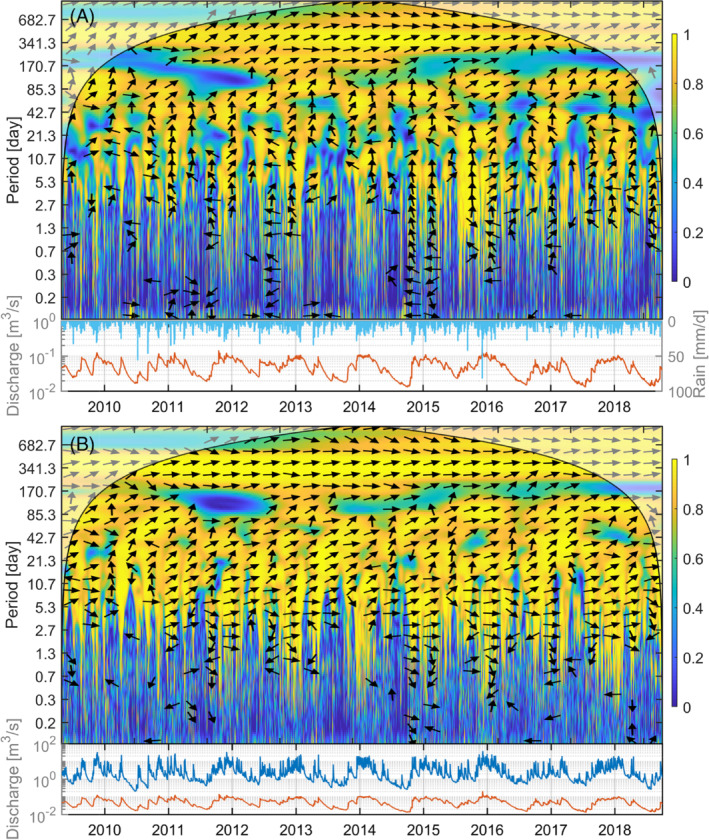
Wavelet coherence (main plots) between (A) hourly rainfall and discharge of Ballindine spring and (B) between hourly discharge of the River Robe and discharge of Ballindine spring between April 29, 2009 and October 1, 2018. The lower plots illustrate the time‐amplitude signals of rainfall (light blue), the spring (red), and the river (dark blue).

The coherence between rainfall and the River Robe and Ballindine spring is plotted in the black windowed area (outside the cone of influence) of Figure 5a and 5b, along with the individual time series on the bottom. The WTC plot illustrates the strength of coherence between frequencies, ranging between 0 (dark blue) and 1 (yellow) at a given time (x‐axis, h) and frequency (y‐axis, y), where 1 indicates a linear relationship. In addition, for high coherence areas the black arrows illustrate the difference in phase ranging between 0 (linear relationship is direct), 180 (linear relationship is indirect) to 360°. Patterns of interest may follow either a horizontal orientation at a given frequency, or a vertical orientation at or during events. A consistent direction of arrows along the y‐axis suggests causality between the input and output variable.

The coherence between Ballindine and rainfall (Figure 5a) suggests a localized and discrete arrangement of patches of high coherence in time and frequency as opposed to a more general background of low coherence: where rain events and discharge surges coincide, a phase‐lock behavior results in high coherence. Overall, three distinct areas of relationship can be identified: (1) Below 10.7 d (256 h) fine strips of coherence interleaved with noncoherent areas representing the alternation of rainfall events (and discharge peaks) and absence of rain and recession periods (even if short). Below 0.7 d (16 h) there is no clear phase relationship as indicated by the varying arrow directions. However, there seems to be a semicontinuous high coherence in the very high‐frequency range at 0.1 d (2 h), which relates to quick responses between rainfall events and discharge peaks; (2) between ∼35 and 72 d (∼842 and 1720 h) high and low coherence areas alternate, presumably linked to an interannual or seasonal pattern. The phase angles generally point upward and increasingly toward the right with increasing period indicating a decreasing time lag; and (3) above 333 d (8000 h) the coherence is very high throughout the entire period being related to the annual or biannual cyclicity. The phase angles are mostly to the right (in‐phase) where the time lag is negligible.

The coherence between Ballindine spring and the River Robe shows a strikingly high coherence throughout frequency and time (Figure 5b), yet strong discontinuities in time are visible for frequencies of 83 to 170 d (2048 to 4096 h). These exceptions are very interesting as it is also present in the coherence plot between the rainfall and Ballindine spring time series (Figure 5a). These low coherences indicate a deviation of linearity between input and output, which is highest during the rainy period of 2012. The frequency range relates to a seasonality, and it is interpreted that the power of spring discharge may be relatively low as compared to the power of the river discharge for the given spectrum. A reason for that may be a physical limitation (transmissivity/effective porosity) of the aquifer, controlling the overall spring discharge, while such limitation is not present for the river discharge. Other possible or additional reasons may be a temporal clogging or filling of conduits as well as the impact of the drainage channels cutting into the topography of the catchment: given a high water level in the aquifer, the upper proportion of the aquifer may be drained directly by the drainage channels, which create an effective “ceiling” to the aquifer, hence, cutting off flow to the spring. Any of these reasons may result in the low coherence at the given spectrum and time.

On the annual/bi‐annual cyclicity (>341 d, >8192 h), the coherence is continuously high, and the frequencies are in phase, which suggests an obvious common annual/biannual periodicity.

Between 341 and 5.3 d (8192 and 128 h), the coherence is still very high, only intersected by locally low coherence. The phase angles range between 0° (in‐phase) and 90° (upward) showing generally a very consistent pattern which is an argument for causality between the frequencies/time series.

The high degree of coherence above ∼5.3 d (>128 h) suggests that the River Robe and Ballindine spring exhibit very similar dynamics, also at low discharge rates. The high coherence may be the result of a similar response to input variables, yet, the consistency of phase angles suggests causality, and hence, clear SW‐GW interaction.

Below frequencies of 5.3 d (128 h), the pattern indicates periods of strong coherence down to 0.3 to 0.7 d (8 to 16 h) during periods of high flow, alternating with periods of strong coherence down to 1.3 to 2.7 d (32 to 64 h) during periods of low flow. Despite the occurrence of high frequency discharge surges of the River Robe during low flow periods, the signal is not transposed towards the spring, and hence, the coherence remains low. Therefore, this seasonal alternation of coherence between 8 to 16 h and 32 to 64 h is interpreted as a change in the impact regime that the river exhibits on the spring. This may be explained by a threshold effect upon which the river impacts on the aquifer, as it will be discussed in the next section.

### Turbidity Sampling

**Figure 6:**
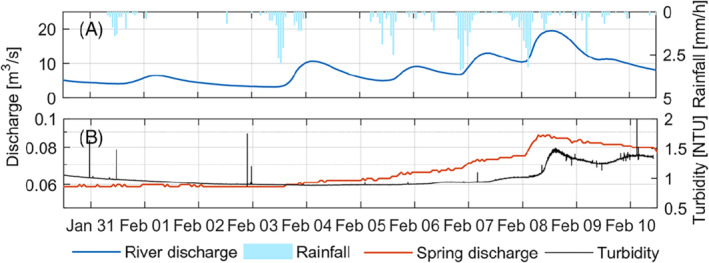
(a) Rainfall and discharge of the River Robe and (b) discharge and turbidity observed at Ballindine spring (*note, turbidity was sampled at 1 min interval—individual peaks may be the result of artifacts*).

Continuous turbidity monitoring was conducted at Ballindine spring to extend the analysis between discharge (head) time series towards a parameter that may indicate transport mechanisms for 10 d (Figure 6).

The discharge of the River Robe shows typical hydrograph to rainfall events including rising limbs and recessions (Figure 6a). The spring discharge at Ballindine exhibited a gradual increase over the period of field observation between January 30, 2019 and February 10, 2019 without any of the fluctuations observed in the river (Figure 6b). However, on February 6 and 8, more discrete spring discharge surges occurred, with the latter one significant in magnitude. These two surges clearly correlate in time with surges of the river flow. In addition, the second surge of spring discharge on February 8 was accompanied by an increase in turbidity. Hence, it is hypothesized that surges in water level relate to pressure transfers and resuspension, mobilizing and/or transporting suspended solids along flow paths between river and the spring. A surge in river discharge >10 m^3^/s (equivalent to >1.66 m in stage) may be considered to be the threshold level for such transport mechanisms.

## Discussion

This study evaluated the temporal dynamics of SW‐GW interaction in conjunction with the means of basic hydrological variables, such as discharge and rainfall. To the knowledge of the authors, it is the first approach using WTC for assessing SW‐GW interactions of karst hydrological systems in time. Given the fact that many SW‐GW studies rely on physicochemical parameters, the approach presented here seems advantageous for an a priori study with only basic data requirements. At the same time, the time series used for this analysis cover at least one hydrological year (or flood and recession periods).

The time‐invariant cross‐correlation demonstrates low linearity between rainfall and spring discharge time series. The relatively gently recessing course of the CCF is commonly interpreted as absence of a quickflow component (Padilla and Pulido‐Bosch 1995). In turn, higher linearity occurs between the river and the spring discharge time series, explaining river bed exfiltration of a losing stream. Similar results were observed by Larocque et al. (1998) who spatially distinguish between the magnitude of exfiltration based on the CCF. The higher linearity between river and spring discharge as opposed to rainfall and spring discharge can be explained by the fact that the spring and river discharge time series are highly autocorrelated, as opposed to the nonautocorrelated rainfall time series. Yet, based on the application of WTC, this study concludes that the difference in linearity between time series is the result of the different impact of input variables (river discharge as opposed to rainfall) onto the aquifer and resulting spring discharge (acknowledging the general uncertainty with regard to cause‐effect interpretations using WTC [Grinsted et al. 2004]). The alternation of high coherence for frequencies larger than 0.3 to 0.7 d during periods of high flow and frequencies larger than 1.3 to 2.7 d during periods of low flow indicates the high temporal resolution of SW‐GW dynamics present. Besides the simple impact of the river on the spring, the power between the frequencies of the river and spring discharge in the form of WTC suggests different dynamics in time and frequency, including a (seasonal) threshold effect upon which the aquifer responds.

Within the given hydrogeological context, a threshold level in the river may activate conduits. While previously, the CCF between rainfall and spring discharge characterized the groundwater flow system as “absent from quickflow,” Larocque et al. (1998) demonstrated the presence of quick flow components using tracer tests, in spite of the CCF between rainfall and spring discharge exhibiting characteristics that would suggest the absence of quickflow (Padilla and Pulido‐Bosch 1995). In fact, this is complemented by the high coherence between rainfall and spring discharge in the very high‐frequency range. However, the hydraulic dynamics are likely to be more complex: in addition to the threshold effect, a ceiling effect was observed, presumably related to the agricultural drainage channels intersecting and draining the top of the aquifer, acting as an upper limitation of groundwater flow in the system. The flow gradient of the drainage channels may change in time, generating a complex and evolving system of infiltration/exfiltration together with the river levels, hence driving recharge, flow and transport mechanisms in this system. This interaction of dynamics can be further investigated by carrying out artificial tracer tests between the river, drainage channels, boreholes (currently none existent) and the spring, along with long‐term physicochemical monitoring of flow within the river and drainage channels, and in the spring discharge. Due to the nature of highly temporal dynamics previously discussed, high‐frequency sampling must be applied (≤1 h).

Below the threshold in the river, the coherence suggests a general muted effect of river on the spring at all times. Changes in SW‐GW dynamics throughout the season may be common (Bailly‐Comte et al. 2009; Chapuis et al. 2020), hence, the WTC technique shows in this study that it is a suitable tool to investigate such a phenomenon. At the same time, the information gained by coherence does not allow any conclusions to be made on the absolute flows between the river and the spring. Yet, such analysis could be conducted by applying numerical modeling of the river and aquifer system coupled with a WTC analysis including, for example, the distinction between periods of linearity vs. nonlinearity (Chinarro et al. 2012), as well as by considering, for example, the use of stable isotopes, focusing on flood events (Binet et al. 2017). Furthermore, such a comprehensive approach can be extended towards the whole spectrum of water levels ranging between low‐flow and high‐flows in order to further investigate a threshold water level upon which the river increases its impact on the aquifer. For example, Binet et al. (2017) conceptualized the time‐variant impact of a losing stream on an aquifer with regard to a threshold water level within an observation well — similar dynamics may occur in the case of the study presented here. Hence, the availability and use of well/borehole data (Bailly‐Comte et al. 2009) between the river and the spring may improve the interpretation of the WTC analysis.

A threshold level in the river flow of 10 m^3^/s, at which an additional impact on the aquifer was observed, was identified through the application of high‐resolution turbidity sampling, which suggested the initiation of transport mechanisms (e.g., resuspension). The occurrence of turbidity in karst systems is highly nonlinear (Beaudeau et al. 2001) and are therefore approached using nonlinear methods (e.g., Savary et al. [2020]). At the same time, turbidity is a valuable indicator for transport mechanisms also in the context of SW‐GW interactions. If available, long‐term turbidity records are likely to support the approach taken in this study, of assessing interactions using the nonlinear method in the form of WTC. Hence, future SW‐GW assessments may beneficially incorporate continuous turbidity sampling.

## Summary

The aim of this research was to investigate SW‐GW dynamics in time between the losing River Robe and the connected outlet of a low‐lying karstified catchment, Ballindine spring, using WTC accompanied by bivariate cross‐correlation using hourly time series of rainfall, stream, and spring discharge for the period of April 29, 2009 to October 1, 2018. The time‐invariant CCF suggests higher linearity between river discharge and spring discharge, than between rainfall and spring discharge. This linearity was interpreted as real influence or causality given the partly very high coherence of frequencies between the river and spring signal as well as the consistency in phases throughout frequencies. The WTC indicates a seasonal pattern in the strength of influence between the river and the spring.

The low coherence in the high‐frequency range during low flow (late recession) periods suggests that the spring does not directly respond to surges of the river water levels. This observation was supported by a threshold effect upon which the river impacts on the aquifer more strongly. Event‐based high‐frequency sampling of turbidity of the spring during multiple rain events supports the interpretation of a threshold level.

This study is an innovative approach that uses time‐variant WTC for the analysis of SW‐GW interactions in karst. The results highlight that more detailed information is gained than applying time‐invariant CCF. No a priori information about any relationships between the observation points is needed, which makes this method suitable as an initial investigation tool on a catchment or even a regional scale.

## Authors' Note

The author(s) does not have any conflicts of interest.
